# Automated IMRT planning with regional optimization using planning scripts

**DOI:** 10.1120/jacmp.v14i1.4052

**Published:** 2013-01-07

**Authors:** Ilma Xhaferllari, Eugene Wong, Karl Bzdusek, Michael Lock, Jeff Z. Chen

**Affiliations:** ^1^ Department of Medical Biophysics University of Western Ontario London Ontario Canada; ^2^ Department of Physics and Astronomy University of Western Ontario London Ontario Canada; ^3^ London Regional Cancer Program London Health Sciences Centre London Ontario Canada; ^4^ Philips Radiation Oncology Systems Fitchburg WI USA; ^5^ Department of Oncology University of Western Ontario and London Health Science Centre London Ontario Canada

**Keywords:** IMRT planning, automated IMRT, head and neck cancer, regional optimization, iterative IMRT optimization

## Abstract

Intensity‐modulated radiation therapy (IMRT) has become a standard technique in radiation therapy for treating different types of cancers. Various class solutions have been developed for simple cases (e.g., localized prostate, whole breast) to generate IMRT plans efficiently. However, for more complex cases (e.g., head and neck, pelvic nodes), it can be time‐consuming for a planner to generate optimized IMRT plans. To generate optimal plans in these more complex cases which generally have multiple target volumes and organs at risk, it is often required to have additional IMRT optimization structures such as dose limiting ring structures, adjust beam geometry, select inverse planning objectives and associated weights, and additional IMRT objectives to reduce cold and hot spots in the dose distribution. These parameters are generally manually adjusted with a repeated trial and error approach during the optimization process. To improve IMRT planning efficiency in these more complex cases, an iterative method that incorporates some of these adjustment processes automatically in a planning script is designed, implemented, and validated. In particular, regional optimization has been implemented in an iterative way to reduce various hot or cold spots during the optimization process that begins with defining and automatic segmentation of hot and cold spots, introducing new objectives and their relative weights into inverse planning, and turn this into an iterative process with termination criteria. The method has been applied to three clinical sites: prostate with pelvic nodes, head and neck, and anal canal cancers, and has shown to reduce IMRT planning time significantly for clinical applications with improved plan quality. The IMRT planning scripts have been used for more than 500 clinical cases.

PACS numbers: 87.55.D, 87.55.de

## I. INTRODUCTION

Intensity‐modulated radiation therapy (IMRT) has become a standard technique in radiation therapy to provide more conformal dose distribution to improve tumor control probability and/or to reduce radiation toxicities. Currently, more than approximately half of every disease sites use IMRT.^(^ ^1^ ^–^ ^4^ ^)^ For some simple cases, such as localized prostate cancer or whole breast irradiation, various class solutions or protocols can be developed to generate an IMRT plan efficiently.^(^ ^5^ ^)^ However, for complicated cases such as some of head and neck cancers, it is still time‐consuming to generate optimized IMRT plans. Besides requirements of accurate delineations of various target volumes and organs at risk (OAR), it is often required to generate additional IMRT optimization structures such as dose limiting ring structures, manually selecting beam directions and energies, IMRT objectives and associated weights. These parameters are generally adjusted manually during the optimization process with trial and error approach, including adding additional IMRT objectives to reduce various cold and hot spots in the dose distribution.

There are on‐going research activities to find more efficient ways for IMRT planning.^(^ ^6^ ^–^ ^12^ ^)^ Multicriteria optimization technique^(^ ^13^ ^–^ ^20^ ^)^ has been introduced into IMRT planning in order to help solve issues faced with single objective planning where a weight for each objective needs to be set before the plan can be optimized. However, currently, it is still time‐consuming with multicriteria optimization to generate and navigate through a large number of plans in Pareto surface. Recently, multicriteria optimization has been commercialized in RaySearch Laboratories' planning system (RaySearch Laboratories, Stockholm, Sweden).^(^ ^21^ ^)^

Regional optimization^(^ ^22^ ^)^ is an effective way to improve IMRT plans by emphasizing specific region of interests to help create high‐dose gradients between target volumes and critical structures during optimization using relatively high importance factors on small region of interests. In this study, we present an iterative method that can be incorporated in clinical process to improve IMRT plan quality and efficiency. Specifically, we have implemented regional optimization in a simple iterative algorithm in a commercial treatment planning system (Pinnacle, Philips Radiation Oncology, Fitchburg, USA). The regional optimization we implemented is based on region of interest (ROI), and is not voxel‐based, as in the original paper.^(^ ^22^ ^)^ Our method is based on automatically generated cold and hot regions in the plan. In this work, we demonstrate that such iterative algorithm is applicable to clinical sites that are generally more challenging in IMRT planning. The method was applied to three clinical sites: head and neck, prostate with pelvic nodes, and anal canal cancers, where we evaluated its efficacies and time savings. In principal, this method can also be used for other sites to automate IMRT planning processes using planning scripts in treatment planning systems.

## II. MATERIALS AND METHODS

### A. Overview

**Figure 1:**
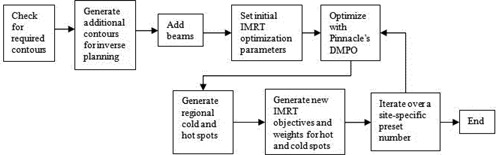
An overview of the steps completed by each script for a clinical site.

For each clinical site, a class solution was first developed manually based on a group of clinical cases. The class solution provides standard beam parameters such as number of beams, their energies, directions, collimator angles, jaw positions, and initial IMRT objectives and corresponding weights. After a class solution was developed for a clinical site, the entire optimization process was incorporated in a planning script. The planning script includes the major activities shown in Fig. 1. For the purpose of providing concrete methodologies, we will explain each of the optimization steps using the National Cancer Institute of Canada Clinical Trials Group head and neck clinical protocol (NCIC CTG HN.6) as an example. This is a good clinical site to illustrate how to automate regional IMRT optimization with many OARs and multiple target volumes. We will also present our evaluation of this technique on two other clinical sites: high risk prostate cancer requiring pelvic nodes radiation and anal canal cancers also with substantial nodal irradiations. Details for automated IMRT planning for these sites are given in the Appendices.

### B. Check required regions of interest

The IMRT scripts require basic regions of interests (ROIs) to be defined, such as all clinical target volumes (CTVs) and all organs at risk (OARs). For example, for the NCIC CTG HN.6, the following ROIs are required: CTV70, CTV63 and/or CTV56, where 70, 63, and 56 are the prescription dose in units of Gray for each volume, with intended doses delivered in 35 fractions. Other required ROIs are: cord, brain_stem, right parotid (rt_parotid), left parotid (lt_parotid), larynx, mandible, rt_cochlea, lt_cochlea, oral_cavity, and one for the body contour (external). Standard nomenclature is required by the script.

It is important to ensure all the required ROIs are present, so that the script can set proper IMRT objectives for these ROIs. The script will check for the required dose matrix and ROIs. If any of the ROIs are missing, it will display names of missing ROIs, in order for a user to add or correct the names of required ROIs. If all the required ROIs are present, the script will set standard colors for ROIs to facilitate quality assurance.

### C. Generate additional contours

After checking for the required ROIs, the iterative algorithm will generate various derived contours such as planning target volume (PTV) for each CTV, planning organ‐at‐risk volumes (PRVs) for required OARs, and various dose‐limiting ring structures for IMRT optimization purpose. For the head and neck IMRT clinical trial, Table 1 gives a summary of all the contours generated.

**Table 1:** Summary of all the contours generated for HN6 clinical trial.

*Contour Name*	*Explanations*	*Contour Name*	*Explanations*
PTV70	Planning target volumes for 70, 63 and 56 Gy prescription doses	TPTV	Total sum of all PTVs
PTV63		cord prv	Planning risk volumes for cord and brainstem with a 5 cm uniform
PTV56		brainstem prv	margin
modPTV70	PTVs that exclude cord_prv, brainstem_prv and not closer to	rt parotid opt	Parotid volumes avoiding PTVs
modPTV63	the external contour by 5 mm	lt parotid opt	
modPTV56		external 5mm	Body contour with a 5 mm margin
optPTV63	Optimization PTVs, avoiding overlap volumes with higher	ring70	1 cm ring around PTV70
optPTV56	prescription doses	ring63	1 cm ring around ring70, and PTV63
optPTV63 m	Optimization PTVs, avoiding overlap volumes with higher	ring56	1 cm ring around ring63, and PTV56
optPTV56 m	prescription doses with a 1 cm margin	ring50	1 cm ring around ring56

The script generates derived ROIs for OARs, such as cord_prv and brainstem_prv with 5 mm margin from cord and brain stem, respectively. Other generated PTVs are modPTV70, modPTV63, and/or modPTV56 that exclude cord_prv, brainstem_prv, and are away from skin by a 5 mm margin. Their purposes are to limit the dose to spinal cord and brain stem to within tolerance and reducing skin dose. Also, it generates optPTV63 and/or optPTV56 that avoids overlapping with higher dose PTVs, such as optPTV63=modPTV63−modPTV70 for optimization purpose. Rings with 1 cm uniform margin around PTVs and/or other rings, such as ring70, ring63, and/or ring56, are created for creating a more conformal dose distribution by specifying a maximum dose in each ring structure. In order to reduce dose to critical structures such as the parotids, rt_parotid_opt and lt_parotid_opt are generated that avoid the PTVs so that more realistic objectives for IMRT optimization can be set. Total sum of the PTVs, TPTV, is generated in order to help define optimal beam geometries.

### D. Add beams

**Figure 2:**
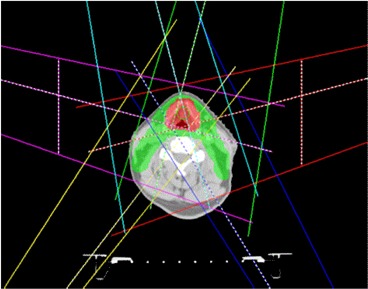
Jaw positions for IMRT fields are fixed in the script to reduce probability of local minimum to avoid beam splitting for more accurate and efficient radiation delivery.

A summary of the six different fields used for HN.6 with their respective couch, gantry, and collimator angles is given in Table 2. Beams are added according to the class solution with fixed jaw sizes based on the PTV coverage and OAR sparing to reduce local minimum problem in IMRT optimization and to improve delivery accuracy and efficiency. TPTV (defined previously) is used to adjust beam geometry that covers the desired volumes. Beam geometry is set by setting the collimator, gantry, and couch angles, and setting the jaw sizes. The jaw sizes for LAO and RAO fields in Table 2 were set to cover the total PTV with 8 mm margin, but it is limited to less than 14.5 cm in order to avoid beam splitting on Varian linacs. Thus, only the side of TPTV where beam direction is along the boundary of TPTV and parotids is made sure to be covered by the fields so that the field edge can provide higher dose gradient between TPTV and parotids. This jaw size is also set to avoid junction of the multileaf collimator (MLC) inside the fields to reduce delivery uncertainty. However, two noncoplanar beams, LSAO and RSAO, are added to cover the whole TPTV with fixed jaw size to avoid beam splitting and to provide dose gradients required for both sides of TPTV. The use of noncoplanar beams is to cover lower neck nodes but avoid irradiation to shoulders. The advantage of using fixed jaw size for large IMRT target volumes in the head and neck was discussed in a recent publication.^(^ ^23^ ^)^ As shown in Fig. 2, two posterior oblique fields cover the PTVs only from one side and shield part of the post neck region with only 2 cm jaw position from central axis for easier MLC segmentation to reduce dose to spinal cord and brain stem.

**Table 2:** Summary of beams generated for HN.6.

	*Gantry Angle*	*Collimator Angle*	*Couch Angle*
LSAO (left superior anterior oblique)	75°	0°	15°
LAO (left anterior oblique)	15°	15°	0°
LPO (left posterior oblique)	148°	350°	0°
RPO (right posterior oblique)	218°	10°	0°
RAO (right anterior oblique)	285°	0°	345°
RSAO (right superior anterior oblique)	345°	350°	0°

After the beam geometry is defined, the proper dose prescription is set. In the case of HN.6, the prescription dose is 70 Gy in 35 fractions to a reference point at the center of GTV. The script will check for the position of the isocenter; this isocenter will be used in all beams. The isodose lines will also be set using standard percentages of prescription with standard colors.

A similar procedure was carried out to implement class solutions (Appendices A and B) for the high risk prostate cancer and anal canal cancer cases.

### E. Initial optimization

IMRT parameters are set in the script, including the maximum number of iterations, the maximum number of control points, minimum segment MU, and area. Then, IMRT objectives and their respective weights are set for the initial optimization using direct machine parameter optimization (DMPO) in Pinnacle. The IMRT objectives used for HN.6 are given in Table 3. Higher weights are given to minimum dose of CTVs and modified or optimization

**Table 3:** Some of the IMRT objectives set for the first optimization for HN.6.

*ROI Name*	*Objective Type*	*Dose (cGy)*	*Weight*
CTV56	Minimum dose	5700	100
CTV63	Minimum dose	6350	100
CTV70	Minimum dose	7100	10
modPTV70	Maximum dose	7690	100
modPTV70	Minimum dose	7000	65
optPTV63	Maximum dose	6720	70
optPTV63	Minimum dose	6450	80
optPTV63_m	Uniform dose	6550	5
optPTV56	Minimum dose	5700	100
optPTV56_m	Maximum dose	6125	100
optPTV56_m	Uniform dose	5650	1
PTV56	Minimum dose	2800	1
PTV63	Minimum dose	3200	1
PTV70	Minimum dose	3000	30
brainstem	Maximum dose	5000	100
brainstem_prv	Maximum dose	5500	33
cord	Maximum dose	4000	100
cord_prv	Maximum dose	4500	70
mandible	Maximum dose	7000	100
lt_parotid_opt	Maximum EUD	2350	20
ring50	Maximum dose	5000	10
ring56	Maximum dose	5600	100
ring63	Maximum dose	6300	50
ring70	Maximum dose	7000	100

PTVs (modPTV70, optPTV63. and optPTV56) to ensure proper dose coverage of CTVs and PTVs that are away from skin by 5 mm. However, we specified lower maximum doses with low weights to the original PTVs to ensure that MLC will open around PTVs, since part of PTVs may be too close or outside patient skin. This will ensure sufficient skin flashing without unnecessary high skin dose. If any CTV is right on the skin, bolus will be used to make sure proper dose coverage. After IMRT objectives are specified, the dose is calculated and the first optimization is then started.

### F. Regional optimization

We implemented the regional optimization to reduce hot and cold spots in IMRT dose distributions automatically in a simple iterative manner.

#### F.1 Generating regional cold and hot spots

After initial IMRT optimization and the final dose calculation using collapsed cone convolution for the first pass, various isodose lines related to the minimum doses to PTVs, maximum doses inside or outside PTVs are converted to contours in the iterative algorithm. Then, the corresponding cold or hot spots in each region are generated, such as in HN.6, cold56, cold63, cold70 for cold spot in optTV56, optPTV63, and modPTV70, respectively. Each cold spot is automatically generated in the script by subtracting the required minimum isodose line (converted to contour) from the target volume. For example, cold70=modPTV70−70 Gy isodose line. Similarly, hot56, hot63, hot70, hot_out_70 is the hot spot in optPTV56, optPTV63, PTV70, and outside PTV70, respectively, and they are generated automatically by the script. For example, hot63=maximum isodose line allowed in PTV63−PTV70−ring70. The reason to subtract higher dose ring ROIs is to avoid conflict with minimum dose coverage of higher dose PTVs. The regional cold and hot spots for HN.6 clinical protocol are listed in Table 4 with their relations to various ROIs and isodose lines. Similar cold and hot spots based on the prescription of each PTV are added for prostate with pelvic nodes and anal canal cases.

**Table 4:** Summary of regional cold and hot spots for HN.6 clinical protocol.

*ROI Name*	*Relation to ROI of Isodose Lines*
hot_out_70	70 Gy isodose line ‐ PTV70 ‐ ring70
hot63	69.3 Gy isodose line ‐ PTV70 ‐ ring70
hot56	61.6 Gy isodose line ‐ PTV70 ‐ PTV63 ‐ ring70 ‐ ring 63
hot70	75.6 Gy isodose line
cold70	modPTV70 ‐ 70 Gy isodose line
cold63	optPTV63 ‐ 63 Gy isodose line
cold56	optPTV56 ‐ 56 Gy isodose line

#### F.2 Iterative optimization with regional cold and hot spots

IMRT objectives for these cold and hot spots are then added for regional optimization in the scripts — for example, objectives listed in Table 5 for HN.6 protocol. The IMRT plan is then continually optimized with these added regional objectives based on previously optimized and segmented plan using DMPO. In this re‐optimization, only MLC segment shape and weights are re‐optimized. The HN.6 script uses 20 iterations. The generation of various hot or cold spots and re‐optimization can be repeated multiple times until an optimal plan is achieved.

**Table 5:** Summary of objectives set for the regional optimization for HN.6 clinical protocol.

*ROI Name*	*Objective Type*	*Dose (cGy)*	*Weight*
hot_out_70	Maximum dose	6800	100
hot63	Maximum dose	6650	50
hot56	Maximum dose	5880	5
hot70	Maximum dose	7350	1
cold70	Minimum dose	7000	100
cold63	Minimum dose	6300	50
cold56	Minimum dose	5600	10

## III. RESULTS

**Figure 3:**
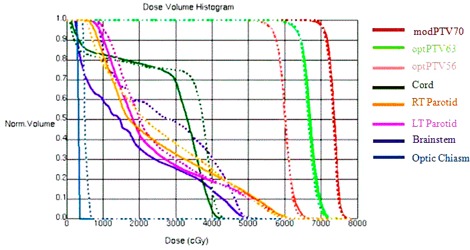
DVH comparison for a head and neck case between manually (dashed line) and automatically generated plans (solid line).

**Figure 4:**
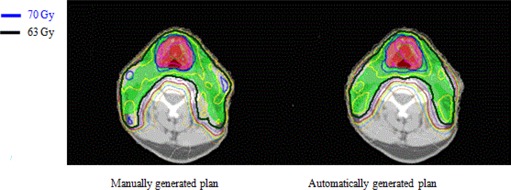
Comparison of dose distribution on a transverse slice for a head and neck case between manually generated IMRT plan and automatically generated IMRT plan using the iterative method. Red shaded volume is PTV70 covered by 70 Gy isodose line in blue, and green shaded volume is PTV63 covered by 63 Gy isodose line in black.

The method has been implemented and tested for three clinical sites: a clinical trial protocol for head and neck cancer, prostate cancer with pelvic nodes, and anal canal cancer. Figure 3 shows DVH comparison for a head and neck case between a previously manually optimized clinical plan and the automatically optimized IMRT plan using the automatic iterative method. A manually optimized plan was used for comparison in this study and the plan was previously optimized by an experienced dosimetrist for clinical use. As shown in Fig. 3, the automatically generated plan has lower dose to the three most sensitive critical structures: brainstem, spinal cord, and the left and right parotids with similar PTV coverage. The comparison of dose distributions on an axial slice is shown in Fig. 4, showing fewer hot spots in the automatically optimized plan.

**Figure 5:**
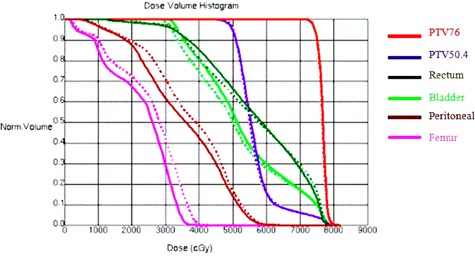
DVH comparison for a prostate case with pelvic nodes irradiation between manually (dashed line) and automatically generated IMRT plans (solid line).

**Figure 6:**
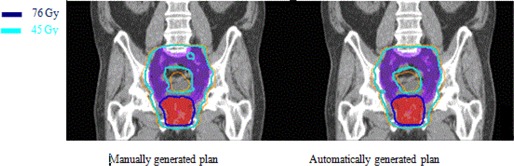
Comparison of dose distribution on a coronal slice for a prostate case with pelvic nodes between manually and automatically generated IMRT plans. Red shaded volume is PTV76 for prostate covered by 76 Gy isodose line shown in dark blue and purple shaded volume is PTV45 for pelvic nodes covered by 45 Gy isodose line shown in light blue.

The results for prostate case with pelvic nodes are shown in Fig. 5 for DVH comparison and Fig. 6 for comparison in dose distributions. It shows that the automatically generated plan is similar to the manually generated clinical plan.

**Figure 7:**
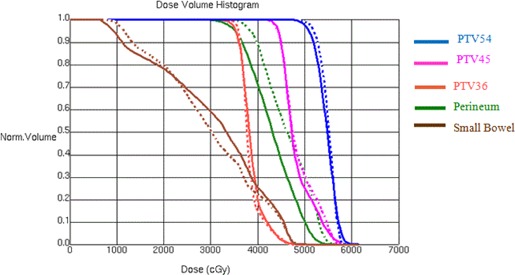
DVH comparison for an anal canal irradiation between manually (dashed line) and automatically generated IMRT plans (solid line).

**Figure 8:**
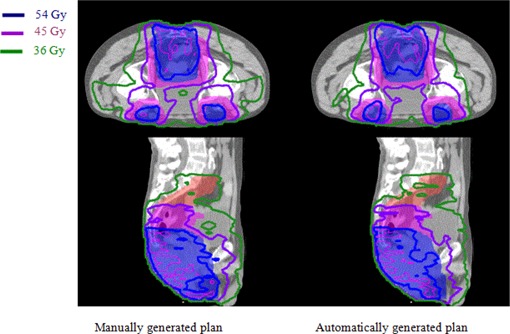
Comparison of dose distributions on a transverse and a sagittal slice for anal canal case between manually and automatically generated IMRT plans. Red, pink, and blue shaded volumes are PTV36, PTV45, and PTV54, respectively, covered by the isodose line 36 Gy shown in green, 45 Gy shown in purple, and 54 Gy shown in blue, respectively.

The results for an anal canal clinical case are shown in Fig. 7 for DVH comparison and Fig. 8 for comparison in dose distributions. It shows automatically generated plan is slightly worse than the manually optimized clinical plan in terms of dose coverage to PTV54 and dose conformality around PTV36.

**Figure 9:**
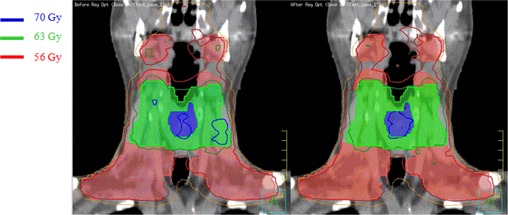
Comparison of dose distribution on the coronal slice for a head and neck case before (left) and after (right) automated regional optimization. Shaded volumes are PTV70 (blue), PTV63 (green), and PTV56 (red), respectively.

As an example, the effect of regional optimization is shown in Fig. 9 for comparison of dose distributions before and after regional optimization using the cold and hot spots for a head and neck case.

The time required to run the planning script on a Pinnacle thin client using computation server with 4 quad core 2.9 GHz CPU is listed in Table 6. The estimated minimum time required for manual planning includes time needed to generate various derived contours, set up beams, dose prescription, manually and repeatedly adjust IMRT objectives, and running IMRT optimization. It shows that the iterative method implemented here as a script can save significant time in IMRT planning. These IMRT planning scripts are used clinically in our center for more than 500 clinical cases since they were implemented clinically in 2010. They include at least 300 head and neck, 180 prostate, and 20 anal canal cases. It should be noted that after using automated planning scripts, most cases will be tweaked by dosimetrists to see if further improvements can be made. However, most tweaking just needs a few more passes of the continuous optimization that usually take less than an hour. The estimated time saved in planning for each case is at least 2 hours for head and neck, and 1 hour for anal canal or prostate with pelvic nodes, depending on the experience of the planner.

**Table 6:** Estimated time required to generate IMRT plans.

	*Manual Planning*	*Automated Planning*
Head and Neck	> 4 hrs	~8 min
Anal Canal	> 2 hrs	~6 min
Prostate with Pelvic Nodes	> 1.5 hrs	~6 min

## IV. DISCUSSION

The present work implemented planning scripts for complex IMRT cases that automated most aspects of plan optimization which otherwise required continual manual input by a planner. We used Pinnacle planning script to automate the regional optimization in an iterative manner; however, the concept can be used in other planning systems, as well. Automated IMRT planning script was recently published for optimization of breast radiotherapy with tangential beams.^(^ ^5^ ^)^ Here, we present an automated planning process for more complicated head and neck, prostate with pelvic nodes, and anal canal cases. It also includes the method to automatically reduce various cold and hot spots in the optimization process. To our knowledge, such implementation for more complex IMRT cases has not been published before. Since the focus of this paper is on the methodology, we only present one example for each clinical site, even though the scripts have been used clinically for more than 500 cases. The thorough statistical analysis of these clinical cases will be presented in a separate paper.

Since many IMRT planning steps are included in our IMRT planning scripts, they generally save many hours in the IMRT planning process. It also helps implement clinical protocols, in‐house standards, using standard dose prescriptions, standard margin for PTVs, standard derived region of interests (ROIs), such as modPTVs, optPTVs, cord_prv, and brainstem_prv, as well as standard color scheme for ROIs and isodose lines. The planning script can reduce variations of plan quality due to different experience of planners. The planning scripts can be improved during clinical use, incorporating new techniques learned in practice.

For many complicated cases, the IMRT planning scripts provide only a good starting point; an optimal plan still requires a planner to fine‐tune the automatically generated plan to adapt the plan for individual situation. Also, the class solution does not consider geometry variations across patients; therefore, it requires fine‐tuning for each individual patient. Such fine‐tuning includes modifying IMRT objectives and their weights. Occasionally, beam parameters may also need to be modified, such as gantry or collimator angles. In future work we will investigate the impact of adjusting IMRT objectives and weights during the iterative optimization process. The regional optimization method presented in this paper may also be combined with priority‐based IMRT optimization method.^(^ ^24^ ^)^

## V. CONCLUSIONS

We have developed IMRT planning scripts for a few clinical sites to automate most of IMRT planning process. In particular, regional optimization has been implemented in an iterative algorithm to reduce hot or cold spots during the optimization process, especially important for complex cases. We demonstrated that this is particularly useful in IMRT planning for head and neck and prostate with pelvic nodes. We have shown that automated iterative inverse planning improves IMRT planning efficiency substantially.

## ACKNOWLEDGMENTS

The authors would like to thank Cancer Care Ontario for funding this work.

## Appendix A. Prostate with pelvic nodes.

In this appendix we give some details on IMRT planning for prostate with pelvic nodes.

The prescription is 76 Gy for the prostate and 50.4 Gy for the pelvic nodes in 35 fractions. The contours generated by the script are listed in Table 7.

**Table 7:** Summary of all the contours generated for prostate with pelvic nodes.

*Contour Name*	*Explanations*	*Contour Name*	*Explanations*
PTV76	Planning target volumes for	TPTV	Total sum of all PTVs
PTV50.4	doses	ring76	1 cm ring around PTV76
optPTV50.4	Optimization PTVs, avoiding overlap volumes with higher prescription doses	ring50.6	1 cm ring around ring76, and PTV50.6
		ring3	1 cm ring around ring50.6

Six IMRT beams were used for IMRT planning, as listed in Table 8. Two of the beams (LAO2 and RAO2) cover both PTVs in order to ensure dose coverage, while the rest of the beams are set at a maximum field size of 14.5 cm to avoid junction of the MLC inside the fields for Varian machines.

**Table 8:** Summary of beams generated for prostate with pelvic nodes.

	*Gantry Angle*	*Collimator Angle*	*Couch Angle*
LAO2 (left anterior oblique 2)	80°	348°	0°
LAO (left anterior oblique)	15°	0°	0°
LPO (left posterior oblique)	135°	340°	0°
RPO (right posterior oblique)	218°	10°	0°
RAO (right anterior oblique)	225°	20°	0°
RAO2 (right anterior oblique 2)	280°	13°	0°

After the contours and the beams are generated, the plan is optimized using the IMRT objectives and weights shown in Table 9.

**Table 9:** Some of the IMRT objectives set for the first optimization for prostate with pelvic nodes.

*ROI Name*	*Objective Type*	*Dose (cGy)*	*Weight*
CTV76	Minimum dose	7600	100
optPTV50_4	Maximum dose	6000	20
PTV50_4	Minimum dose	5040	40
PTV76	Uniform dose	7650	5
PTV76	Minimum dose	7600	35
Bladder	Maximum EUD	4500	1
Bladder	Maximum dose	7550	10
Bowel	Maximum dose	5040	100
Femur_LT	Maximum dose	4000	10
Femur_RT	Maximum dose	4000	10
Rectum	Maximum dose	7500	10
Rectum	Maximum EUD	4500	1
ring76	Maximum dose	7500	1
ring50.4	Maximum dose	5000	1
ring3	Maximum dose	4000	2

After the first pass of IMRT optimization, the cold and hot spots are generated automatically, as displayed in Table 10.

**Table 10:** Summary of regional cold and hot spots for prostate with pelvic nodes.

*ROI Name*	*Relation to ROI of Isodose Lines*
max79	80 Gy isodose line
ptv_cold	PTV76 ‐ 72.2 Gy isodose line

The IMRT plan will be continually optimized with the new contours with the IMRT objectives listed in Table 11.

**Table 11:** Summary of objectives set for the regional optimization for prostate with pelvic nodes.

*ROI Name*	*Objective Type*	*Dose (cGy)*	*Weight*
max78	Maximum dose	7900	40
ptv_cold	Minimum dose	7350	40

## Appendix B. Anal canal.

There are multiple IMRT planning scripts generated for anal cancer with a total of ten different variations based on the stage, number of target volumes, and different prescriptions. There are minor deviations between each script due to different treatment types. As an example, this Appendix gives some details for the situation with dose prescription of 54 Gy to the primary tumor volume, 45 Gy, and 36 Gy to different nodal volumes given in 30 fractions.

After checking required contours, the automated IMRT script generates additional contours, as shown in Table 12.

**Table 12:** Summary of all the contours generated for anal canal cancer case.

*Contour Name*	*Explanations*	*Contour Name*	*Explanations*
PTV54	Planning target volumes for 54, 45 and 36 Gy	TPTV	Total sum of all PTVs
PTV45	prescription doses	external 5mm	Body contour with a 5 mm margin
PTV36			
PTV54 eval	PTVs that exclude the external contour by 5 mm	small bowel opt	Normal structure volumes avoiding PTVs
PTV45 eval		Bladder opt	
PTV36 eval		Perineum opt	
ptv45 eval M	Optimization PTVs, avoiding overlap volumes with higher	ring54	1 cm ring around PTV54
ptv36 eval M	prescription doses and rings	ring45	1 cm ring around ring54, and PTV45
ptv45 eval only	Optimization PTVs, avoiding overlap volumes with higher prescription doses	ring36	1 cm ring around ring45, and PTV36

Six beams were used for IMRT planning. as listed in Table 13. Two of the posterior beams (LPO2 and RPO2) cover all the target volume, while the rest of the beams have a maximum field size of 14.5 cm.

**Table 13:** Summary of beams generated for anal canal.

	*Gantry Angle*	*Collimator Angle*	*Couch Angle*
LAO (left anterior oblique)	195°	10°	0°
LPO (left posterior oblique)	335°	0°	0°
LPO2 (left posterior oblique 2)	280°	0°	0°
RPO (right posterior oblique)	25°	0°	0°
RPO2 (right posterior oblique 2)	75°	0°	0°
RAO (right anterior oblique)	165°	350°	0°

Some of the IMRT objectives used by the plan automation are shown in Table 14.

**Table 14:** Some of the IMRT objectives set for the first optimization for case of anal canal cancer.

*ROI Name*	*Objective Type*	*Dose (cGy)*	*Weight*
GTV	Uniform dose	5450	1
PTV54_eval	Minimum dose	5400	80
PTV54_eval	Maximum dose	5600	40
PTV54_eval	Uniform dose	5500	10
PTV45_eval	Minimum dose	4550	80
ptv45_eval_M	Uniform dose	4600	40
ptv45_eval_M	Maximum dose	4900	100
ptv45_eval_only	Maximum dose	5300	5
PTV36_eval	Minimum dose	3600	80
ptv36_eval_M	Maximum dose	3900	100
ptv36_eval_M	Uniform dose	3700	5
Bladder_opt	Maximum dose	4300	50
Perineum_opt	Maximum dose	4300	10
Perineum_opt	Maximum EUD	4000	50
small_bowel_opt	Maximum dose	4500	15
small_bowel_opt	Maximum EUD	2800	1
Femur_RT	Maximum dose	4700	1
Femur_LT	Maximum dose	4700	1
right54	Maximum dose	5400	10
ring45	Maximum dose	4500	10
ring36	Maximum dose	3600	5

After the first pass of IMRT optimization, cold and hot spots were generated using the isodose lines, as shown in Table 15.

**Table 15:** Summary of regional cold and hot spots for anal canal cancer case.

*ROI Name*	*Relation to ROI of Isodose Lines*
hotspots	54 Gy isodose line ‐ PTV54_eval ‐ ring54
hot54	58 Gy isodose line
hot45	49.5 Gy isodose line ‐ PTV54_eval ‐ ring54
hot36	39.6 Gy isodose line ‐ PTV45_eval ‐ PTV54_eval ‐ ring54 ‐ ring45
cold36	PTV36_eval ‐ 36 Gy isodose line
cold45	PTV45_eval ‐ 45 Gy isodose line
cold54	PTV54_eval ‐ 54 Gy isodose line

IMRT objectives were added for these hot and cold spots, as shown in Table 16.

**Table 16:** Summary of final objectives set for the regional optimization for anal canal cancer.

*ROI Name*	*Objective Type*	*Dose (cGy)*	*Weight*
hot54	Maximum dose	5670	10
hot45	Maximum dose	4700	1
hot36	Maximum dose	3780	1
hotspots	Maximum dose	4800	2
cold54	Minimum dose	5400	35
cold45	Minimum dose	4500	60
cold36	Minimum dose	3600	20
